# Supplement and fermented food use among older US adults with dermatologic disorders: A cross-sectional analysis

**DOI:** 10.1016/j.jdin.2026.09.016

**Published:** 2026-10-07

**Authors:** Miriam Motlak, Hyoungsun Park, Dongwon Lee, Gerardo Guilarte, Sonal Choudhary, Thomas K. Le

**Affiliations:** Department of Dermatology, University of Pittsburgh Medical Center, Pittsburgh, Pennsylvania

**Keywords:** alopecia areata, complementary and alternative medicine, eczema, fermented foods, integrative dermatology, probiotics, psoriasis, skin infections, supplements, vitiligo

To the Editor:

Given growing patient interest in integrative care, dermatology providers are more frequently asked to offer evidence-based guidance on supplement and fermented food intake.^1^ Emerging evidence suggests that gut microbiome modulation can support barrier function and reduce inflammation.^2^ Probiotics have shown benefits for inflammatory skin disorders, including acne and psoriasis.^2^ Given limited data on current usage patterns among patients with dermatologic issues, we aimed to examine associations between supplement and fermented food use and several dermatologic disorders in a real-world population.

We analyzed data from the 2019 Health and Retirement Study, a nationally representative cohort of US adults aged 50 years and older, because it uniquely captures both self-reported health conditions and special food consumption in a longitudinal, nationally weighted sample. This cross-sectional analysis examined self-reported intake of supplements and fermented foods, stratified into 5 binary domains (Supplementary Table 1, available via Mendeley at https://data.mendeley.com/datasets/3fhrsszd7h/2). Outcomes included self-reported psoriasis, eczema, vitiligo, alopecia areata, and skin infections (ie, cellulitis). Survey weights generated population-level estimates. Multivariable logistic regression analyses yielded odds ratios (ORs) adjusted for sociodemographic factors, including age, sex, race, ethnicity, education, and insurance coverage. Prevalence estimates included respondents with complete data on all 5 skin condition variables (n = 4648).

This analysis included data from 5097 respondents with a mean survey weight of 15,572, corresponding to approximately 79.4 million US adults. Baseline sociodemographic characteristics and supplement/fermented food intake patterns were stratified by dermatologic condition (Table I). Regular consumption of any fermented food or supplement was significantly associated with psoriasis (OR 1.56, *P* = .04), eczema (OR 1.54, *P* = .01), and skin infections (OR 1.63, *P* = .02) (Fig 1). In category-specific analyses, probiotic use was associated with eczema (OR 2.06, *P* = .001) and skin infections (OR 1.88, *P* = .005). Similarly, dairy-based fermented foods were associated with eczema (OR 1.54, *P* = .02) and skin infections (OR 1.60, *P* = .03). Pickled food consumption was associated with skin infections (OR 2.73, *P* = .02). No significant associations were observed for plant-based fermented foods or fermented beverages, and no exposure category was significantly linked to vitiligo or alopecia areata.

**Table I:** Demographic characteristics and supplement and fermented food intake patterns among individuals with psoriasis, vitiligo, eczema, alopecia areata, and skin infections (ie, cellulitis) compared with those without those conditions

Characteristics	Patients with psoriasis, n (%)	Patients without psoriasis, n (%)	*P*∗	Patients with vitiligo, n (%)	Patients without vitiligo, n (%)	*P*∗	Patients with alopecia areata, n (%)	Patients without alopecia areata, n (%)	*P*∗	Patients with eczema, n (%)	Patients without eczema, n (%)	*P*∗	Patients with skin infections, n (%)	Patients without skin infections, n (%)	*P*∗
**Age**			.294			.154			.789			.044			.050
<60	16 (8.5)	283 (6.2)		8 (6.5)	290 (6.3)		1 (2.0)	296 (6.3)		33 (8.6)	265 (6.0)		10 (4.7)	290 (6.4)	
60-69	76 (40.4)	1855 (40.5)		39 (31.7)	1892 (40.8)		22 (44.9)	1910 (40.6)		168 (43.6)	1763 (40.2)		76 (35.5)	1852 (40.7)	
70-79	57 (30.3)	1371 (29.9)		39 (31.7)	1384 (29.8)		14 (28.6)	1402 (29.8)		114 (29.6)	1316 (30.0)		65 (30.4)	1355 (29.8)	
80-89	37 (19.7)	906 (19.8)		29 (23.6)	913 (19.7)		10 (20.4)	932 (19.8)		61 (15.8)	884 (20.1)		49 (22.9)	897 (19.7)	
90+	2 (1.1)	166 (3.6)		8 (6.5)	160 (3.4)		2 (4.1)	163 (3.5)		9 (2.3)	161 (3.7)		14 (6.5)	151 (3.3)	
**Gender**			.778			.012			.002			<.001			.421
Male	74 (39.4)	1863 (40.7)		36 (29.3)	1897 (40.9)		9 (18.4)	1921 (40.8)		116 (30.1)	1814 (41.3)		81 (37.9)	1857 (40.9)	
Female	114 (60.6)	2718 (59.3)		87 (70.7)	2742 (59.1)		40 (81.6)	2782 (59.2)		269 (69.9)	2575 (58.7)		133 (62.1)	2688 (59.1)	
**Race**			<.001			.499			.769			.478			.002
White	154 (81.9)	3451 (75.3)		99 (80.5)	3502 (75.5)		35 (71.4)	3557 (75.6)		302 (78.4)	3310 (75.4)		185 (86.4)	3411 (75.0)	
Black	12 (6.4)	801 (17.5)		15 (12.2)	798 (17.2)		11 (22.4)	798 (17.0)		60 (15.6)	750 (17.1)		22 (10.3)	790 (17.4)	
Other	22 (11.7)	321 (7.0)		9 (7.3)	331 (7.1)		3 (6.1)	340 (7.2)		23 (6.0)	321 (7.3)		7 (3.3)	336 (7.4)	
Unknown	0 (0.0)	8 (0.2)		0 (0.0)	8 (0.2)		0 (0.0)	8 (0.2)		0 (0.0)	8 (0.2)		0 (0.0)	8 (0.2)	
**Hispanic**			.948			.464			.236			<.001			.001
Unknown	0 (0.0)	2 (0.0)		0 (0.0)	3 (0.1)		0 (0.0)	3 (0.1)		1 (0.3)	2 (0.0)		1 (0.5)	2 (0.0)	
Mexican	14 (7.4)	347 (7.6)		10 (8.1)	353 (7.6)		0 (0.0)	367 (7.8)		8 (2.1)	354 (8.1)		4 (1.9)	362 (8.0)	
Other Hispanic	10 (5.3)	206 (4.5)		9 (7.3)	204 (4.4)		2 (4.1)	209 (4.4)		15 (3.9)	202 (4.6)		7 (3.3)	205 (4.5)	
Non-Hispanic	164 (87.2)	4026 (87.9)		104 (84.6)	4079 (87.9)		47 (95.9)	4124 (87.7)		361 (93.8)	3831 (87.3)		202 (94.4)	3976 (87.5)	
**Education**			.371			.028			.695			<.001			.002
Less than high school	15 (8.0)	598 (13.1)		19 (15.4)	595 (12.8)		3 (6.1)	610 (13.0)		22 (5.7)	593 (13.5)		11 (5.1)	606 (13.3)	
GED	6 (3.2)	220 (4.8)		7 (5.7)	218 (4.7)		3 (6.1)	220 (4.7)		15 (3.9)	211 (4.8)		12 (5.6)	210 (4.6)	
High school diploma	94 (50.0)	2175 (47.5)		71 (57.7)	2193 (47.3)		25 (51.0)	2235 (47.5)		178 (46.2)	2096 (47.8)		104 (48.6)	2157 (47.5)	
Associate degree	12 (6.4)	279 (6.1)		6 (4.9)	286 (6.2)		4 (8.2)	288 (6.1)		33 (8.6)	261 (5.9)		11 (5.1)	282 (6.2)	
Bachelor's degree	37 (19.7)	715 (15.6)		9 (7.3)	741 (16.0)		7 (14.3)	745 (15.8)		68 (17.7)	682 (15.5)		36 (16.8)	711 (15.6)	
Master's degree	18 (9.6)	404 (8.8)		4 (3.3)	417 (9.0)		4 (8.2)	412 (8.8)		51 (13.2)	370 (8.4)		26 (12.1)	397 (8.7)	
Professional degree	5 (2.7)	137 (3.0)		6 (4.9)	137 (3.0)		3 (6.1)	139 (3.0)		16 (4.2)	124 (2.8)		13 (6.1)	129 (2.8)	
Unknown	1 (0.5)	53 (1.2)		1 (0.8)	52 (1.1)		0 (0.0)	54 (1.1)		2 (0.5)	52 (1.2)		1 (0.5)	53 (1.2)	
**Insurance**			.562			1.000			1.000			.286			.017
No	15 (8.0)	436 (9.5)		12 (9.8)	438 (9.4)		5 (10.2)	449 (9.5)		30 (7.8)	421 (9.6)		10 (4.7)	447 (9.8)	
Yes	173 (92.0)	4145 (90.5)		111 (90.2)	4201 (90.6)		44 (89.8)	4254 (90.5)		355 (92.2)	3968 (90.4)		204 (95.3)	4098 (90.2)	
**Any supplement or fermented food**			.037			.175			.003			<.001			.003
No	103 (54.8)	2867 (62.6)		69 (56.1)	2900 (62.5)		20 (40.8)	2938 (62.5)		197 (51.2)	2780 (63.3)		112 (52.3)	2853 (62.8)	
Yes	85 (45.2)	1714 (37.4)		54 (43.9)	1739 (37.5)		29 (59.2)	1765 (37.5)		188 (48.8)	1609 (36.7)		102 (47.7)	1692 (37.2)	
**Probiotics**			.054			.911			.789			<.001			<.001
No	144 (76.6)	3773 (82.4)		100 (81.3)	3809 (82.1)		39 (79.6)	3861 (82.1)		270 (70.1)	3651 (83.2)		155 (72.4)	3754 (82.6)	
Yes	44 (23.4)	808 (17.6)		23 (18.7)	830 (17.9)		10 (20.4)	842 (17.9)		115 (29.9)	738 (16.8)		59 (27.6)	791 (17.4)	
**Dairy-based fermented**			.607			.082			.430			<.001			.004
No	138 (73.4)	3451 (75.3)		84 (68.3)	3505 (75.6)		34 (69.4)	3542 (75.3)		258 (67.0)	3337 (76.0)		143 (66.8)	3439 (75.7)	
Yes	50 (26.6)	1130 (24.7)		39 (31.7)	1134 (24.4)		15 (30.6)	1161 (24.7)		127 (33.0)	1052 (24.0)		71 (33.2)	1106 (24.3)	
**Plant-based fermented**			.410			.437			.574			.167			.013
No	175 (93.1)	4340 (94.7)		114 (92.7)	4393 (94.7)		45 (91.8)	4453 (94.7)		358 (93.0)	4160 (94.8)		194 (90.7)	4309 (94.8)	
Yes	13 (6.9)	241 (5.3)		9 (7.3)	246 (5.3)		4 (8.2)	250 (5.3)		27 (7.0)	229 (5.2)		20 (9.3)	236 (5.2)	
**Fermented beverages**			.712			.294			.064			.138			.839
No	159 (84.6)	3931 (85.8)		101 (82.1)	3984 (85.9)		37 (75.5)	4037 (85.8)		320 (83.1)	3775 (86.0)		182 (85.0)	3899 (85.8)	
Yes	29 (15.4)	650 (14.2)		22 (17.9)	655 (14.1)		12 (24.5)	666 (14.2)		65 (16.9)	614 (14.0)		32 (15.0)	646 (14.2)	
**Pickled foods**			.278			.185			.361			.610			.017
No	176 (93.6)	4378 (95.6)		114 (92.7)	4435 (95.6)		45 (91.8)	4495 (95.6)		365 (94.8)	4192 (95.5)		197 (92.1)	4351 (95.7)	
Yes	12 (6.4)	203 (4.4)		9 (7.3)	204 (4.4)		4 (8.2)	208 (4.4)		20 (5.2)	197 (4.5)		17 (7.9)	194 (4.3)	

∗ *P* values were calculated using chi-squared tests.

**Fig 1:**
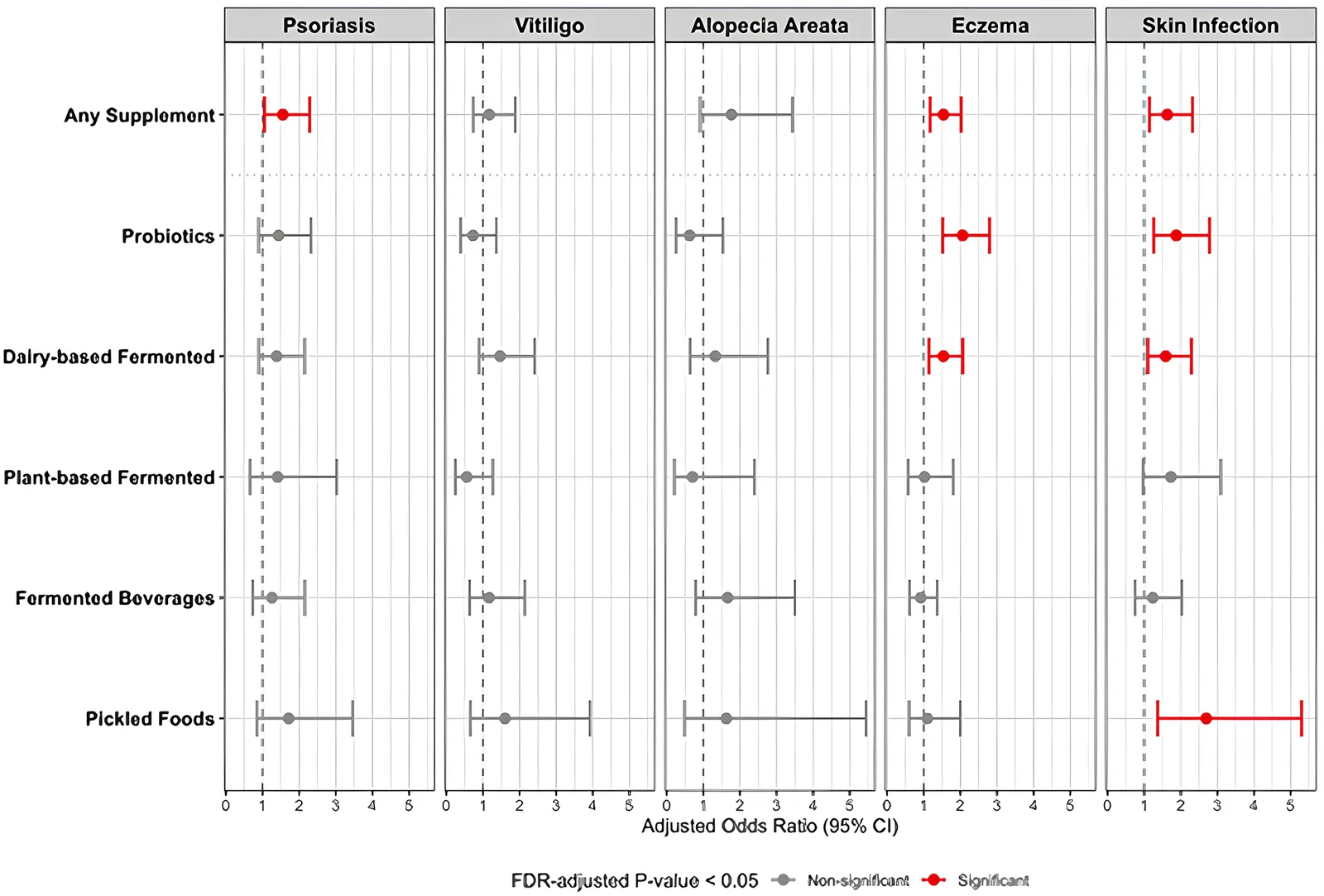
Association of supplement and fermented food intake with dermatologic conditions. Odds ratios adjusted for age, sex, race, ethnicity, education, and insurance coverage. The Benjamini-Hochberg correction was applied for multiple comparisons within each exposure category.

Our findings align with other population-level studies, showing a higher prevalence of supplement use among patients with chronic health conditions.^3^ Patients with inflammatory skin disorders may initiate supplements for multiple reasons, including to promote skin health.^4^ Specific health-seeking behaviors can also influence dietary intake; for example, patients with skin infections may consume pickled foods, which have demonstrated antimicrobial and antioxidant properties.^5^

Limitations include the cross-sectional nature of the Health and Retirement Study survey; reverse causation and confounding by health-seeking behaviors are possible, as patients with chronic skin disorders may be more likely to initiate supplements following diagnosis. Other possible limitations include exposure and outcome misclassification. Therefore, findings should be interpreted as descriptive associations, rather than evidence of benefit, harm, or biologic effect. The Health and Retirement Study also primarily samples older adults, limiting generalizability to younger populations. Nevertheless, dermatology providers should routinely ask patients about their intake of supplements and fermented products and offer guidance on potential benefits, medication interactions, and risks.

## Conflicts of interest

None disclosed.
